# Temperature-Dependent Modulation of Cardiac Metabolism, Post-Injury Survival and Regenerative Rate in Axolotls

**DOI:** 10.3390/metabo16060414

**Published:** 2026-06-13

**Authors:** Anita Dittrich, Sofie Amalie Andersson, Aage Kristian Olsen Alstrup, Pernille Lajer Sørensen, Mette Irene Theilgaard Simonsen, Maibritt Hald Arildsen, Rasmus Roost Aabling, Henrik Lauridsen

**Affiliations:** 1 Comparative Medicine Lab, Department of Clinical Medicine, Aarhus University, 8200 Aarhus, Denmarkpernillelajer@biomed.au.dk (P.L.S.); rasaab@clin.au.dk (R.R.A.); 2 Department of Nuclear Medicine, Aarhus University, 8200 Aarhus, Denmark; aagealst@rm.dk (A.K.O.A.);; 3 Department of Clinical Medicine, Aarhus University, 8200 Aarhus, Denmark

**Keywords:** metabolism, heart regeneration, PET, respirometry, ectotherm, *Ambystoma mexicanum*

## Abstract

**Background/Objectives**: Cardiac regenerative ability varies in vertebrates. Adult mammals cannot mount a regenerative response, while fetal mammals and some salamanders and teleosts fully regenerate the heart after a cryoinjury mimicking a myocardial infarction. This contrast is suggested to be regulated in part by metabolism, with high regenerative capacity correlating with a comparatively lower mass-specific metabolic rate, ectothermy rather than endothermy and a metabolic phenotype favoring glycolysis in cardiac muscle. **Methods**: In this physiological study on axolotl salamanders, we altered the housing temperatures from the standard 20 °C to 10 °C, 25 °C and 30 °C and assayed key metabolic parameters as well as cardiac function, survival and regenerative capacity. **Results**: Our study demonstrated that while axolotls could be housed at temperatures ranging from 10 °C to 30 °C in an uninjured state, signs of a pathological response involving cardiac and metabolic insufficiency and mortality, especially after cryoinjury, increased progressively with increasing temperatures. We observed several metabolic effects, including differences in oxygen consumption, plasma metabolites and cardiac function. Cardiac regeneration after cryoinjury progressed as expected with only a small remaining injury after 60 days at the standard housing temperature of 20 °C. Regeneration was highly reduced in a reversible manner at 10 °C while regenerative rate was not affected at 25 °C. At 30 °C, cardiac regeneration could not be evaluated as the majority of animals (five out of six) did not survive the injury, likely reflecting insufficient cardiac reserve capacity to simultaneously sustain thermal metabolic effects and support tissue repair. **Conclusions**: The ectothermic axolotl undergoes several metabolic changes when exposed to different housing temperatures, with heart regeneration showing a narrower permissive temperature range than survival of the axolotl in an uninjured state.

## 1. Introduction

The axolotl salamander is a prominent model organism for cardiac regeneration, due to its ability to undergo functional and anatomical regeneration after an injury such as cryoinjury [1,2]. The fundamental biological limitations of cardiac regeneration are still unclear, but a comparatively low mass-specific metabolic rate, cardiomyocytes with an immature metabolic phenotype, and ectothermy rather than endothermy are proposed to be some of the main traits associated with high regenerative ability across vertebrate species [3,4,5].

Because metabolism is regulated by environmental temperature in ectotherms like the axolotl, altering housing temperature regulates energy demand because the rate of biochemical reactions is subject to the ambient temperature in an animal that cannot independently regulate their body temperature [6,7,8,9,10,11]. Generally, any ectothermic species can live within a temperature range of a critical minimum temperature (CT_min_) and a critical maximum temperature (CT_max_), in which metabolic turnover, performance, growth, homeostatic processes and aging progress faster with increasing temperature until an optimal temperature (T_opt_) point, before eventually reaching a lethal temperature (T_lethal_) [6,12]. Importantly, this relationship can be different for individual tissues and processes.

The mechanisms underlying CT_max_ and T_lethal_ are generally believed to be underpinned by breakdown of optimal function at two levels: (1) the subcellular level via loss of function of critical enzymes and proteins as reviewed elsewhere [7,13] or (2) higher-order systems like the cardiovascular system, which is the focus of this study [12,14,15]. Cryoinjury would be expected to functionally reduce CT_max_ and T_lethal_ on a systems level [16]. Importantly, acclimatization can, to some degree, extend the temperature range [17,18,19,20,21,22]. Limb regeneration has been shown to progress faster at higher temperatures in newts [23]. However, cardiac injury may pose a unique challenge because increased cardiac function is a fundamental requirement to sustain higher metabolic demands, and any loss of cardiac tissue at higher temperatures may therefore be lethal. In addition, we have previously demonstrated how adaptations in cardiac metabolism are part of the regenerative response in the axolotl [24], similarly to what has been reported in neonatal mice and zebrafish, where metabolic reprogramming has been shown to be required for cardiomyocyte dedifferentiation and proliferation [25,26,27], meaning that metabolic adaptations to temperature changes may alter the balance of pro- versus anti-regenerative metabolic phenotypes.

The aim of the study was to investigate how axolotl metabolism and cardiac physiology are affected by a range of survivable temperatures, and to determine the temperature range in which cardiac regeneration is possible in this regenerative species. We hypothesized that there would be a temperature range, narrower than the CT_min_ to CT_max_, in which cardiac regeneration could occur because decreasing temperatures would slow down metabolic turnover and/or vital processes at the subcellular level, while increasing temperatures would possibly speed up the regenerative process, like after limb amputation [23]. On the other hand, due to the vital function of the heart and increased overall metabolic demand, the loss of cardiac reserve capacity after injury may become detrimental at higher temperatures, either resulting in immediate mortality or reducing the capacity for regeneration by affecting the heart’s ability to allocate resources towards anabolic regeneration rather than catabolic energy turnover.

## 2. Materials and Methods

### 2.1. Animal Experiments

**Figure 1:**
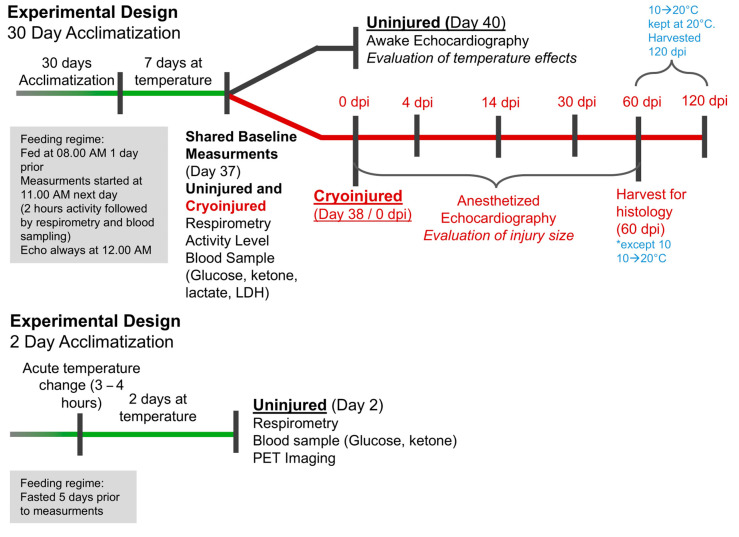
Experimental Design. Two different acclimatization regimens were used as illustrated in the flowchart. For the 30-day acclimatization, the number of animals used for shared baseline measurements on day 37 were: 10 °C (*n* = 10), 20 °C (*n* = 10), 25 °C (*n* = 8) and 30 °C (*n* = 12). Animals were then allocated to uninjured (and awake echocardiography) or cryoinjured (and anesthetized echocardiography + histology) groups such that the numbers of both uninjured and injured animals were *n* = 5 for 10 °C, *n* = 5 for 20 °C, *n* = 4 for 25 °C and *n* = 6 for 30 °C. The 10 → 20 °C group was only used for cryoinjury and anesthetized echocardiography + histology. For the acute exposure, all animals were uninjured and used for all measurements with *n* = 6 for each temperature. Dpi = days post-injury.

In total, 66 animals (both sexes, all sexually mature adults, 45.66 ± 18.74 g body mass) were used for this study. Animals were acquired from a private Danish breeder or the Department of Zoophysiology at Aarhus University, Denmark. They were housed in individual plastic crates (39 cm × 28 cm, 10 cm water depth) on a 12 h/12 h light/dark cycle. Groups were housed at four different housing temperatures, 10 °C, 20 °C, 25 °C and 30 °C, after acclimatization for 2 days for acute exposure or 30 days for prolonged/complete acclimatization (Figure 1). Furthermore, one group was initially kept at 10 °C for sixty days post-injury and then transferred to 20 °C for an additional sixty days. No animals that were still alive at the time of measurement or any collected data were excluded from data analysis or reporting of our results.

To obtain the different housing temperatures, the facility was kept at the standard 20 °C. Animals housed at 10 °C were kept in a glass-front fridge, while animals housed at 25 °C and 30 °C were gradually warmed to the appropriate temperature by placing their crate into a larger plastic container heated by an aquarium heater. Temperature was monitored routinely throughout the experiment. For 30-day-acclimatized experiments, all groups that were not kept at the standard 20 °C were gradually acclimatized to their experimental temperature over a 30-day period and maintained at their set temperature for 1 week prior to echocardiography, blood sampling, and injury The housing temperature was either increased by 1 °C/6 days for animals housed at 25 °C or increased/decreased by 1 °C/3 day for animals housed at 10 °C and 30 °C. For acute-response experiments, animals in the housing crate were moved from 20 °C to the experimental temperature, reaching the target temperature within a few hours, and maintained for two days prior to experiments (Figure 1).

All animals exposed to the 30-day acclimatization protocol were used for shared baseline measurements of oxygen consumption (respirometry), activity levels and blood sampling (glucose, ketones, lactate and LDH) before being assigned to either an uninjured group (and awake echocardiography for evaluation of temperature effects) or a cryoinjured group (and anesthetized echocardiography for evaluation of injury size + histology). Injury size is more easily measured under anesthesia with benzocaine because this increases contractility, making the injury more apparent (while masking temperature-specific effects).

Axolotls were fed axolotl pellets three times weekly (Axobalance from, Aquaterratec, Bröckel, Germany). To ensure even availability of food between groups, feeding was normalized to an expected Q_10_ of ∼2.2 [28], such that 10 °C animals were provided with two pellets, 20 °C animals four pellets, 25 °C animals six pellets and 30 °C animals eight pellets at each feeding. The 30-day-acclimatized animals were always fed in the morning one day prior and then assayed in the afternoon the next day. The 2-day-acclimatized animals were fasted for five days prior to respirometry and blood sampling and another day before PET imaging in order to avoid the specific dynamic action response [29].

Humane endpoints were defined as a loss of more than 20% body mass, refusing food for more than one week or animals being non-responsive to tail pinch. Animals were euthanized while under benzocaine anesthesia by exsanguination (which naturally follows the collection of the heart) followed by decapitation.

Prior to surgery, blood sampling and non-awake echocardiography, animals were anesthetized for 30 min by submersion in 0.2% *w*/*v* benzocaine. For acute-response experiments and PET imaging, animals were instead anesthetized by 15 min of submersion in 35 mg/L propofol (Propofol B, Braun, Melsungen, Germany). Cardiac injury was induced using a cryoprobe as previously described [2]. In brief, the ventricle was exposed and a copper probe cooled in liquid nitrogen was placed onto the ventroapical face of the heart for 10 s.

We prioritized using animals from the same batch rather than size-matching across batches throughout the experiments, so long as all animals could be determined as sexually mature adults (evident by body shape and keratinized toe tips). Importantly, the mass-specific oxygen consumption rate of axolotls begins to plateau at a body mass of around 30 g (Supplementary Figure S1).

All animal experiments were done in accordance with the national Danish legislation for care and use of laboratory animals and 2010/63/EU and approved by the Danish National Animal Experiments Inspectorate (protocol # 2020-15-0201-00688, valid 28 December 2020 until 28 December 2025, with all experiments herein being performed within this time frame).

### 2.2. Echocardiography

Echocardiography was performed on both awake (to assay cardiac function at different temperatures in uninjured animals) and anesthetized axolotls (to assay regenerative progress in cryoinjured animals). Regenerative progress in terms of injury size was evaluated prior to injury at 0 days post-injury (dpi) as well as at 4, 14, 30, and 60 dpi (with additional subsequent time points for the 10 °C → 20 °C group that was kept at 20 °C for an additional 60 days and finally ended at 120 dpi). During anesthesia with benzocaine, axolotls were placed in the supine position, just covered with water and the transducer was lowered directly down onto the chest of the axolotl. For awake imaging, animals were placed in a plastic hammock and allowed to settle for 15 min, before the ultrasound transducer, covered with ultrasound-gel, was gently placed against the chest from underneath, taking care not to disturb the animals. Imaging and downstream analysis was performed as previously described with the Visualsonics Vevo 2100 ultrasound system (version 1.7, https://www.visualsonics.com) [2]. Long-axis views of the ventricle and infarction, as well as Doppler, were used to determine heart rate and ventricular and infarction area. All measurements were obtained using the Vevo Lab software as means across three cardiac cycles. Heart rate was measured directly by Doppler in the Vevo lab software and tracings were made around the ventricle at maximum diastole and maximum systole, as well as the non-contracting area (injury) at maximum diastole. Volumes at diastole and systole were calculated assuming a spherical ventricular geometry using the equation

$$V=\frac{4}{3}{\sqrt{\frac{area}{\pi }}}^{3}$$

VFAC was then calculated as

$$VFAC=\frac{V_{Diastole\;}-V_{Systole}\;}{V_{Diastole}}$$

and mass-specific cardiac output as

$$CO=\frac{Stroke\;Volume\;\left(V_{Diastole\;}-V_{Systole}\right)\times Heart\;Rate}{Body\;Mass\;}$$

Non-contracting fraction was calculated as

$$NCF=\frac{V_{non-contracting}}{V_{Diastole}}\times 100$$

### 2.3. Respirometry

Uninjured axolotls were placed in a respirometry chamber (1 L glass container with airtight lid). The container was filled completely with aerated water (20.95% O_2_) and all air bubbles were removed by filling the container through a narrow channel in the lid using a 50 mL syringe. Oxygen content was measured using a Unisense OXY-meter (Unisense Scientific, Aarhus, Denmark) at the start and end of incubation in the chamber. The duration of incubation in the chamber was dependent on housing temperature to ensure no animals experienced hypoxia during respirometry (120 min at 10 °C, 90 min at 20 °C, 75 min at 25 °C and 60 min at 30 °C). O_2_ consumption was normalized to the time between measurements, specific mass of water in the container and animal body mass.

### 2.4. Activity Tracking

Uninjured animals were placed in their normal housing crates with enrichments removed on a uniform blue surface. A regular smartphone was installed on a tripod system to capture a time-lapse video for ∼1 h (2 frames per s) after an initial 1 h of acclimatization. Activity tracking was always performed between 11 a.m. and 12 p.m. (one day and three hours after last feeding). The first 10 min were discarded to account for any behavioral effects as a result of people entering the room to begin the recording. During analysis the videos were played back at 4× speed, and a stopwatch was used to measure the total time during which the animal was moving around to obtain a % active time measurement. Time spent “paddling” towards the edges of the crate without moving forward was counted as active time.

### 2.5. PET-Imaging

Uninjured and anesthetized animals were injected with [18F]-FDG (~25 MBq/animal) intravenously in the jugular vein and allowed 2 h of circulation time at their set housing temperature (with or without aeration). [18F]-FDG is a glucose analog that cannot be metabolized by the cells, and thus, accumulation of FDG is a proxy for glucose uptake. The appropriate circulation time for FDG to reach steady-state for PET imaging has been previously determined in axolotls [30]. Combined PET and MRI imaging was obtained on a Mediso NanoScan PET/MRI system (Mediso, Budapest, Hungary) to detect [18F] decay with an isotropic image resolution of 0.4 mm and an acquisition time of 20 min for PET and an additional 20 min for MRI. The axolotls were wrapped in moist paper towels soaked in anesthetic fluid throughout the 40 min scan and immediately returned to housing water afterwards. Signal values were measured within regions of interest in the different tissues using ImageJ (version 1.54a) and reported as values relative to the signal in the brain.

### 2.6. Blood Metabolite Measurements

For glucose and ketone measurements, blood samples were collected from benzocaine-anesthetized axolotls from the gill arch using a 31 G 0.5 mL syringe pre-coated with heparin. A single drop of whole blood was placed onto each strip (FreeStyle Precision Neo Glucose strips and ketone strips (Abbott Diabetes Care Inc., Alameda, CA, USA)) and assayed in a commercial glucometer (Freestyle Precision Neo). The instrument reports both glucose and ketone values to the first decimal point and is reported to have a level of accuracy that fulfills the current ISO 15197:2013 criteria (global standards for trusted goods and services set by the International Organization for Standardization (ISO)) [31].

For plasma lactate measurements, blood samples were centrifuged at 3000 *g* for 5 min and the plasma was collected, transferred to cryovials, snap-frozen in liquid nitrogen and stored at −80 °C until all samples had been collected over the course of 6 months. The plasma samples were thawed on ice, gently mixed by pipetting and diluted in assay buffer (5 µL sample to 95 µL assay buffer). The diluted samples were loaded in a clear 96-well plate and assayed along with a standard reference in serial dilution using a commercial colorimetric kit according to the respective manufacturer’s protocol (Sigma Aldrich, St. Louis, MO, USA. Cat.nr: MAK065). Absorbance was analyzed at 450 nm on a Biochrome ELISA plate reader (Biochrom Ltd., Cambridge, UK).

### 2.7. LDH Microplate Assay

Tissue damage was measured in plasma using the CyQUANT LDH cytotoxicity assay (Invitrogen, Carlsbad, CA, USA. Cat.nr: C20300). Plasma samples were diluted 1:9 in ultrapure water and 50 µL samples were loaded in a clear 96-well plate along with a positive control included in the kit. Then, 50 µL reaction mix was added and the plate was incubated protected from light for 1 h before addition of 50 µL stop solution, and the absorbance was measured at 490 nm on a Biochrom EZ Eliza plate reader (Biochrom Ltd., Cambridge UK).

### 2.8. Collection of Hearts

Hearts were collected for histology at 60 dpi (or 60 + 60 dpi in the case of the animals transferred from 10 °C to 20 °C) by embedding in cryomolds in M-Freeze embedding medium (Sigma Aldrich, St. Louis, MO, USA. Cat.nr: 103693). The molds were frozen by placing them on a Styrofoam platform floating in liquid nitrogen for 30 min before moving them to −80 °C for storage before sectioning. All hearts were cryosectioned at −20 °C in series, at 8 µm thickness with 125 µm distance between each section. The fresh frozen sections were then stored at −20 °C for about one week prior to staining.

### 2.9. Immunofluorescence

Cryosections were fixed in 4% PFA for 15 min, followed by 20 min permeabilization in 0.25% Tritron-X and 3 h blocking with a combination of 5% goat serum, 3% BSA and 0.1% Tritron-X in 70% PBS. After blocking, sections were incubated with primary antibody (anti α-actinin (Invitrogen, Carlsbad, CA, USA. Cat.nr: MA1-22863)) overnight at 4 °C, washed with 70% PBS, incubated for 4 h at room temperature with secondary antibody (Goat anti-mouse IgG Alexa Fluor 647 (Invitrogen, Carlsbad, CA, USA. Cat.nr: A-21240)) and washed again with 70% PBS. Sections were then stained with WGA (wheat germ agglutinin coupled to Alexa Fluor 488 (Invitrogen. Carlsbad, CA, USA. Cat.nr: W11261)) for 1 h at room temperature at a concentration of 10 µg/mL in 70% PBS. Finally, nuclei were stained with Hoechst for 30 min (Invitrogen. Carlsbad, CA, USA. Cat. nr: H21486) at a concentration of 2 µg/mL, washed and mounted with ProLong Diamond anti-fade mounting media (Invitrogen. Carlsbad, CA, USA. Cat.nr: P36965).

All the stained sections (16–20 sections per heart) were imaged on an upright widefield fluorescence slide-scanner (Olympus V2120 (Olympus Corporation, Tokyo, Japan)) at 20× magnification. Analysis was done in Qpath by superimposing a point grid with a 500 µm distance between the points. Each point within the ventricle was characterized as either (1) healthy myocardium, (2) remaining injury or (3) empty space, which was not included in calculating the infarction fraction. An example of an analyzed section is shown in Supplementary Figure S2.

### 2.10. Statistics

Graphs and statistics were done in GraphPad Prism version 10.2.0 for Windows (GraphPad Software, Boston, MA, USA, www.graphpad.com) in all cases except for the PET image analysis, which was done in Fiji ImageJ (version 1.54a) [32] and Microsoft Excel with the RealStatistics expansion pack (Resource Pack software (Release 8.9.1). Copyright (2013–2023) Charles Zaiontz. www.real-statistics.com). When comparing the different groups at a single time point, analysis was performed by one-way ANOVA and Dunnett’s post hoc tests for multiple comparison of all other groups to the 20 °C control group. Exceptions were made in cases where the standard deviations were significantly different (*p* < 0.05), in which case the Brown–Forsythe and Welch ANOVA and Dunnett’s post hoc test were used instead. In the case of one-way ANOVA, normal distribution was ensured using the Shapiro–Wilk test.

When comparing groups across time, two-way ANOVA (time, temperature) with repeated measures and Dunnett’s post hoc tests were performed to test for statistically significant differences within all groups compared to a starting time (baseline in all cases except the non-contracting fraction, where 4 dpi was considered the starting time where the injury was at maximum size). For this purpose, only animals that survived until 60 dpi were included in the analysis in order to perform repeated-measures analysis. However, when testing for significant differences between groups across several time points, a mixed-effects analysis with Šidák post hoc tests was performed in order to include animals that did not survive until the endpoint, again, comparing all other groups to the 20 °C control group.

## 3. Results

### 3.1. Axolotls Could Tolerate Housing Temperatures as High as 30 °C After a 30-Day Acclimatization, but Post-Injury Mortality Was Increased with Higher Temperatures

Although the recommended housing temperature for axolotls is ∼18–20 °C [33], we found that axolotls could tolerate housing temperatures as high as 30 °C after gradual acclimatization for 30 days (acclimatization can increase T_lethal_ in salamanders [34]).

**Figure 2:**
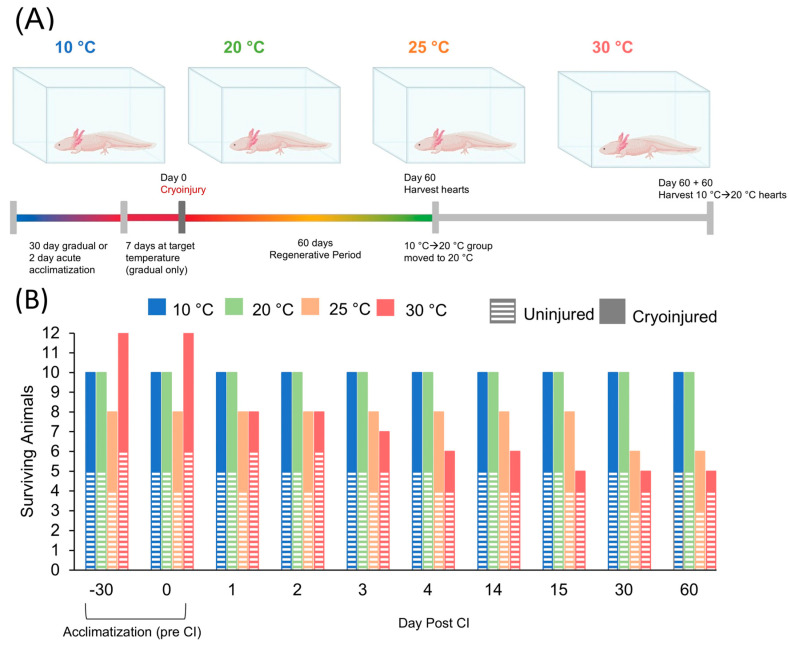
Experimental set-up and survival. (**A**) Axolotls were kept in housing water with different temperatures, with 20 °C being the standard housing temperature. Animals were either acclimatized gradually over 30 days, followed by 7 days at target temperature, or acutely over 2 days. Cryoinjury was performed on day 38 (0 days post-injury) and hearts were harvested 60 days after injury (or 60 + 60 days in the case of the 10 °C → 20 °C group that was moved at day 60). (**B**) Survival in the experimental groups across the regenerative period with uninjured groups in striated bars and cryoinjured animals in solid bars (only relates to the 30-day acclimatization; mortality was 0% in the acute exposure with only 2 days of acclimatization). No statistical test was performed for survival.

During the acclimatization phase and subsequent shared baseline measurements, survival in all groups (uninjured and cryoinjured collectively) was 100%. After cryoinjury (CI), survival among cryoinjured groups was still 100% in the 10 °C and 20 °C groups, while survival dropped to 75% 30 dpi (days post-injury) in the 25 °C group, with a single animal in both the uninjured and CI groups dying. More noticeably, survival in the 30 °C group dropped to 66% 1 dpi. While all the uninjured 30 °C animals were still alive at 1 dpi, only two out of six injured animals awoke from post-operative anesthesia, and only a single injured animal in the 30 °C group survived until 60 dpi (Figure 2B). Animals that were acclimatized more acutely for two days had a 100% survival rate and were not included in Figure 2B.

### 3.2. After 30 Days of Acclimatization, O_2_ Consumption Rate Was Affected but Did Not Scale with Temperature in Uninjured Animals

**Figure 3:**
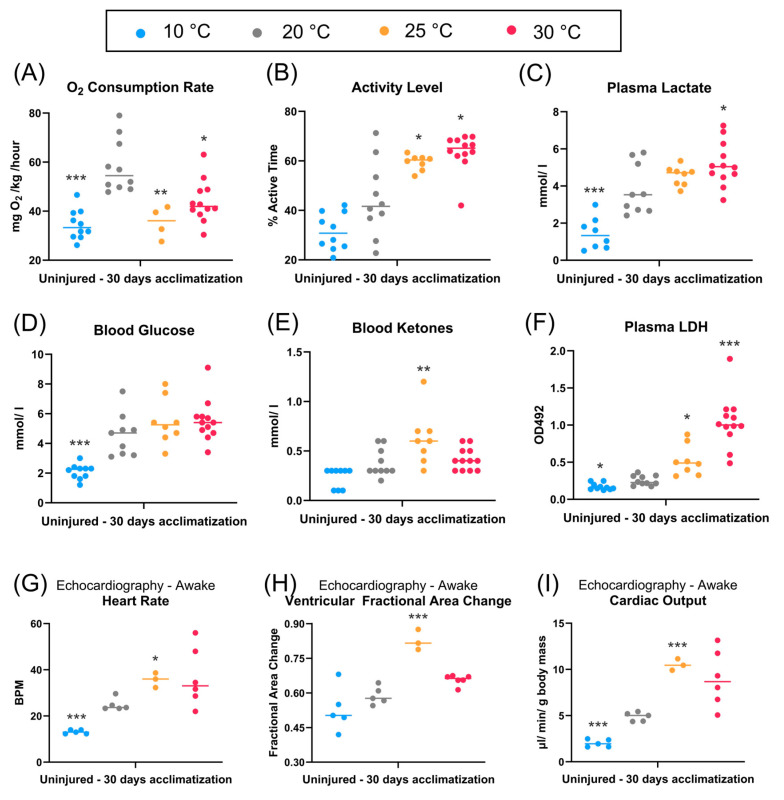
Metabolic adaptations to different housing temperatures in uninjured animals after 30 days of acclimatization. (**A**) O_2_ consumption during closed respirometry in awake animals. (**B**) Activity levels at different housing temperatures as a percentage of time spent moving in an undisturbed setting. (**C**) Plasma lactate concentrations measured by microplate assay. (**D**) Blood glucose and (**E**) ketones measured using a glucometer on fresh whole blood samples. (**F**) Plasma values of LDH as a biomarker of tissue damage expressed as absorption values at 492 nm in a microplate assay. (**G**) Heart rate, (**H**) ventricular fractional area change (VFAC) and (**I**) cardiac output calculated as an average of three cardiac cycles from echocardiography performed in awake animals. Data presented in (**A**–**F**) are shared baseline measurements before allocation into uninjured and cryoinjured groups with *n* = 10 for 10 °C, *n* = 10 for 20 °C, *n* = 8 for 25 °C and *n* = 12 for 30 °C. Awake echocardiography in (**G**–**I**) was performed on animals allocated to the uninjured groups with *n* = 5 for 10 °C, *n* = 5 for 20 °C, *n* = 4 for 25 °C and *n* = 6 for 30 °C. Asterix indicates a significant difference in a group from the 20 °C control group: * = *p* < 0.05, ** = *p* < 0.005 and *** = *p* < 0.0005. Statistical testing was done with one-way ANOVA with Dunnett’s post hoc test with multiple comparisons made against the 20 °C control group, except in cases of unequal standard deviations among the groups, where the Brown–Forsythe and Welch variation of ANOVA was instead used (applies to (**B**,**F**,**G**,**I**)).

Using closed respirometry, we found that O_2_ consumption rate (aerobic metabolism) was affected by housing temperature (One-way ANOVA and Dunnett’s post hoc test compared to 20 °C control). O_2_ consumption rate was significantly decreased at a lower housing temperature of 10 °C (34.52 ± 5.81 mg O_2_/kg/h) compared to 20 °C (58.34 ± 10.38 mg O_2_/kg/h) (*p* < 0.0001). Surprisingly, an inverse effect was not seen at higher temperatures. In fact, O_2_ consumption rate in the 25 °C group was significantly lower compared to the 20 °C group (35.38 ± 3.85 mg O_2_/kg/h) (*p* = 0.0002). In the 30 °C group, O_2_ consumption rate was also significantly lower than the 20 °C group at 44.82 ± 8.19 mg O_2_/kg/h (*p* = 0.0012) (Figure 3A).

To ascertain if these shifts in O_2_ consumption rates were a reflection of increased activity levels, we measured the percentage of active time the animals spent in an undisturbed setting at the four different housing temperatures. We found that, unlike O_2_ consumption rate, activity levels scaled with temperature (Brown–Forsythe and Welch ANOVA with Dunnett’s post hoc tests) (Figure 3B). Mean percentage activity levels were 31.57 ± 7.01% at 10 °C, 44.40 ± 14.25% at 20 °C, 59.41 ± 2.86 at 25 °C and 63.58 ± 7.20% at 30 °C. This was statistically significantly different from 20 °C at 25 °C and 30 °C (*p* = 0.0328 and *p* = 0.0082 respectively). Thus, activity levels could not explain the observed differences in O_2_ consumption rate. Importantly, O_2_ consumption cannot be equated to metabolic rate as it does not factor in anaerobic metabolism.

### 3.3. Changes in Plasma Lactate, Blood Glucose and Ketones After 30 Days of Temperature Acclimatization

Plasma lactate was significantly lower compared to the 20 °C control (3.83 ± 1.28 mmol/L) in the 10 °C group (1.44 ± 0.80 mmol/L) and higher in the 30 °C group (5.76 ± 0.88 mmol/L) (one-way ANOVA with Dunnett´s post hoc test, *p* = 0.0002 (10 °C group) and *p* = 0.0178 (30 °C group)) (Figure 2C). This may indicate an increase in anerobic metabolism with increasing temperatures, which at least in part could explain how increased metabolic demand was not met by increased O_2_ consumption (Figure 2A). Blood glucose was significantly lower in the 10 °C group with 2.09 ± 0.47 mmol/L compared to 4.58 ± 1.35 mmol/L in the 20 °C control group (one-way ANOVA with Dunnett´s post hoc test, *p* = 0.0004) (Figure 2D). Blood ketones were higher only in the 25 °C group at 0.62 ± 0.25 mmol/L (one-way ANOVA with Dunnett´s post hoc test, *p* = 0.0080) (Figure 2E).

### 3.4. Higher Housing Temperatures Were Associated with Elevated Tissue Damage After 30 Days of Acclimatization

To assay whether metabolic changes with higher temperatures could in part be connected to an adverse pathological effect, we measured the level of lactate dehydrogenase (LDH) in plasma samples from uninjured animals after 30 days of temperature acclimatization (Figure 2F). LDH is a general biomarker of cell/ tissue damage [35]. We found an overall effect of increasing plasma LDH at higher temperatures (Brown-Forsythe and Welch ANOVA with Dunnett´s post hoc tests compared against the 20 °C control group). The OD absorbance value at 492 nm from a microplate LDH assay was 0.17 ± 0.04 in the 10 °C group, 0.25 ± 0.06 in the 20 °C group, 0.52 ± 0.19 in the 25 °C group and 1.04 ± 0.33 in the 30 °C group. This represented a statistically significant decrease compared to 20 °C in the 10 °C group (*p* = 0.0147), and an increase in the 25 °C (*p* = 0.0206) and 30 °C groups (*p* < 0.0001), indicating a connection between temperature and tissue damage.

### 3.5. Cardiac Function Was Altered by Temperature After 30 Days of Acclimatization in Uninjured and Awake Axolotls

To assay cardiac function as an effect of temperature without the known effects of anesthesia [36], we performed echocardiography on uninjured and awake axolotls after 30 days of temperature acclimatization (Brown–Forsythe and Welch ANOVA with Dunnett’s post hoc tests (heart rate and cardiac output) or one-way ANOVA with Dunnett’s post hoc test (VFAC) comparing against the 20 °C control group). Axolotls housed at 10 °C adapted mainly via a reduction in heart rate (Figure 3G), leading to reduced cardiac output (Figure 3I) without significantly affecting contractility measured as fractional ventricular area change (VFAC) (Figure 3H) (*p* = 0.0006 (heart rate), *p* = 0.4071 (VFAC) and *p* < 0.0001 (cardiac output)). The animals housed at 20 °C had an average heart rate of 24.9 ± 2.41 BPM, VFAC of 0.58 ± 0.03 and a cardiac output of 4.88 ± 0.43 µL/min/g body mass. For 10 °C animals, the corresponding values were 13.13 ± 0.78 BPM, 0.52 ± 0.08 VFAC, and 2.01 ± 0.36 µL/min/g.

When increasing housing temperature to 25 °C, cardiac output was increased to 10.80 ± 0.51 µL/min/g body mass (*p* = 0.0005) (Figure 3I), but in this case via an increase in both heart rate which rose significantly to 35.66 ± 2.59 BPM (*p* = 0.0206) (Figure 3G), and contractility (VFAC), which was elevated to 0.80 ± 0.03 (*p* = 0.0001) (Figure 3H).

When increasing the temperature further to 30 °C, the heart rate presented with large variations (41.83 ± 9.59 BPM) and was not statistically different from the 20 °C control (Figure 3G). VFAC was measured at 0.65 ± 0.02, which was also not statistically different from the 20 °C control (Figure 3H). As a result, cardiac output showed a large standard deviation and no statistically significant difference from the control at 9.81 ± 2.78 µL/min/g body mass. Behaviorally, we observed that animals were more responsive to touch and less likely to lie still during awake echocardiography at higher temperatures, which likely contributed to the large variance in heart rate in the 30 °C group. The lack of proportional increase in cardiac function at 30 °C along with elevated plasma LDH could indicate a pathological response associated with some degree of cardiac insufficiency, which is a likely primary driver of increased mortality, possibly in combination with metabolic insufficiency.

### 3.6. Acute Exposure to Different Housing Temperatures Led to a Successive Increase in Oxygen Consumption with Higher Temperatures in Uninjured Animals

**Figure 4:**
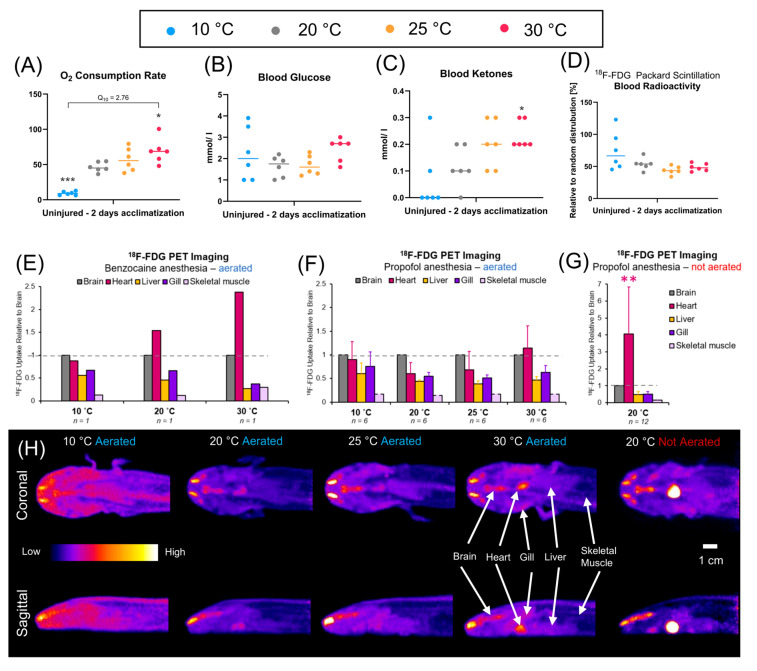
Metabolic effects of acute exposure to different temperatures in uninjured axolotls in vivo. (**A**) Oxygen consumption assayed by closed respirometry. (**B**) Blood glucose and (**C**) ketones measured with glucometer. (**D**) Blood radioactivity after 2 h of circulation with [18F]-FDG. (**E**) [18F]-FDG uptake in different tissues relative to the brain in pilot animals in aerated water anesthetized with benzocaine. (**F**) [18F]-FDG uptake in different tissues relative to the brain in animals anesthetized with propofol in aerated water. (**G**) [18F]-FDG uptake in different tissues relative to the brain in animals anesthetized with propofol in non-aerated water. (**H**) Coronal (top row) and sagittal (bottom row) maximum intensity projections of positron emission tomography images of [18F]-FDG (glucose analog) distribution after 2 h circulation time with propofol anesthesia at 10 °C, 20 °C, 25 °C, and 30 °C in aerated and non-aerated conditions. Animals used for blood sampling (**B**,**C**) were on the same day used for PET imaging under propofol anesthesia (**F**) with *n* = 6 for each temperature. The same batch of animals was used for the experiments shown in (**E**,**G**) but with ample time in between experiments in order to completely wash out anesthesia and return to standard temperature. Error bars represent standard deviation. Asterisk indicates significant differences: * = *p* < 0.05, ** = *p* < 0.005 and *** = *p* < 0.005. In the case of O_2_ consumption rate, multiple comparison of all other groups compared to the 20 °C control was done by one-way ANOVA with Dunnett’s post hoc tests. For glucose, ketones and blood radioactivity, the same tests were done instead with Brown–Forsythe and Welch ANOVA due to unequal standard deviations among the groups. For PET imaging tests were done with one-way or two-way ANOVA (depending on whether different temperatures were involved) with Tukey post hoc tests comparing all tissues and temperatures when applicable.

Next, we wanted to test if the metabolic changes at different temperatures were dependent on the 30-day acclimatization period and if the animals were functionally capable of scaling their oxygen consumption and thus aerobic metabolism according to the higher temperatures, at least in the short term. We therefore exposed axolotls to a more acute effect by gradually cooling or warming their housing water for a few hours, exposing them to a change in temperature for only two days prior to measuring O_2_ consumption. The acute exposure to different temperatures led to a significant increase in O_2_ consumption rate with progressively higher temperatures (one-way ANOVA with Dunnett’s post hoc tests against the 20 °C control: *p* = 0.0002 (10 °C), *p* = 0.3525 (25 °C) and *p* = 0.0135 (30 °C)) (Figure 4A). Ectotherms have limited abilities to offset the increased metabolic demand associated with higher temperatures. In the absence of behavioral thermoregulation this leaves the possibility of a shift towards anaerobic metabolism after longer acclimatization (supported by elevated plasma lactate) and/or thermal acclimatization involving changes in enzyme expression and mitochondrial capacity (possibly pathological). Importantly, the oxygen consumption levels should only be directly compared between different temperatures within the same acclimatization regime, as the feeding regimen was not the same during the 2-day versus 30-day acclimatization and a host of confounding physiological effects may be in effect that do not allow for a direct comparison between acute and prolonged changes in housing temperature.

### 3.7. Blood Ketone, but Not Glucose, Levels Were Affected After Acute Exposure to Different Temperatures in Uninjured Animals

Whereas blood glucose levels were not significantly different after two days of acute exposure to different temperatures in uninjured animals (Figure 4B), blood ketone levels were significantly different between 20 °C and 30 °C, although in general there were large variations within all groups (Figure 4C) (Brown–Forsythe and Welch ANOVA with Dunnett’s post hoc tests, *p* = 0.0339). These results indicated that the decreased glucose levels observed at 10 °C after 30 days of acclimatization were, like O_2_ consumption rate, an effect that only materialized after a longer acclimatization period. In contrast, elevated ketones at higher temperatures appeared to manifest more quickly. This could indicate that in the short term, the expected higher metabolic demand at higher temperatures was met in part by aerobic metabolism of fat (leading to elevated plasma ketones) (Figure 3E). Importantly, values should not be compared directly between these two regimes, but only between temperature groups on the same feeding regime.

### 3.8. The Increased Demand on the Heart in Response to Increased Temperatures Was Not Met by Increased Uptake of Glucose in Uninjured Animals

To further elucidate whether the demands on the heart could be the defining factor in the difference in tolerance to cryoinjury at different temperatures, we performed PET imaging upon administration of the radioactive glucose analog [18F]-FDG after two days of acute exposure in uninjured animals. A pilot experiment (*n* = 1) indicated that whereas glucose uptake in the brain and in the heart was similar at 10 °C, the heart experienced a greatly upregulated intake relative to the brain at increased temperatures (Figure 4E). When repeating the experiment with a larger batch of animals (*n* = 6 in a paired set-up, i.e., the same six animals at different temperatures), we opted to use propofol since previous studies have revealed a strong stimulatory effect of benzocaine on the axolotl cardiovascular system [36].

The observation of the pilot experiment was not supported with propofol anesthesia, suggesting that the cardiac effect seen initially could be an anesthetic effect. Glucose uptake in the brain and heart was not significantly different at any temperature (two-way ANOVA (brain vs. heart, *p* = 0.76) and (different temperatures, *p* = 0.11) (Figure 3H and Figure 4F). Taken together with a non-significant difference in glucose uptake at higher temperatures evident by the radioactivity of blood samples relative to random distribution (Figure 4D) but also with the much elevated O_2_ consumption rate in the same range of temperatures (Figure 4A), these results showed that following an acute exposure to different temperatures (in a state of fasting but non-starving and in well-aerated water), glucose metabolism did not play the major role in meeting the expected globally increased metabolic demand nor the local metabolic need of the heart relative to a well-known great consumer of glucose such as the brain at higher temperatures.

In both the pilot experiment using benzocaine and the propofol experiment, we bubbled the housing water during the [18F]-FDG circulation time because the administration of the radiotracer into the jugular vein requires anesthetized axolotls, which could compromise O_2_ uptake and induce hypoxia. Since glucose is the key in anaerobic metabolism, we also tested glucose uptake by repeating the propofol experiment at 20 °C without bubbling the housing water. In contrast to the previous propofol experiment, this led to a heavily increased [18F]-FDG uptake by the heart relative to the brain (one-way ANOVA with Tukey post hoc test, *p* = 0.0010) (Figure 4G,H), demonstrating that while glucose uptake in the axolotl heart at higher water oxygen levels is relatively unaffected by temperature, it is highly dependent on the availability of oxygen. Importantly, the level of dissolved oxygen in water is temperature-dependent, with 100% O_2_ saturation at 20 °C equating to 9.1 mg/L, while at 30 °C it drops to 7.55 mg/L, meaning animals housed at warmer temperatures will be in water lower in oxygen.

### 3.9. Heart Regeneration After Cryoinjury Progressed at a Lower Rate at Colder Temperatures but Was Not Affected by Survivable Higher Temperatures

**Figure 5:**
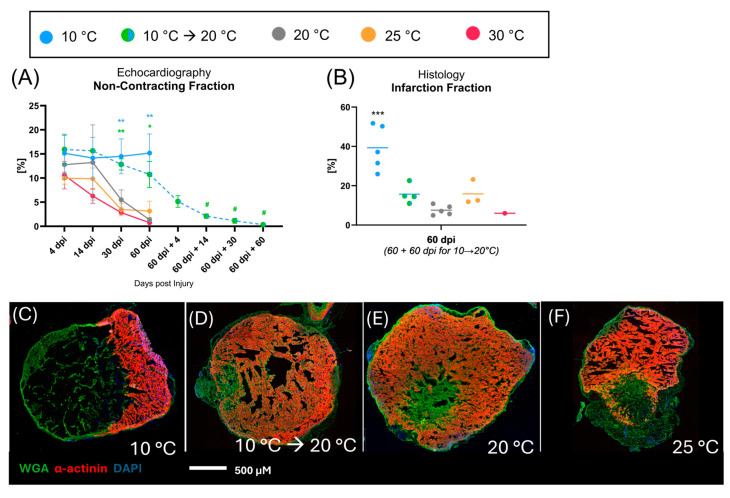
Regeneration of cryoinjury at different temperatures in vivo. (**A**) Non-contracting fraction measured by echocardiography. All datapoints are an average across three cardiac cycles and statistical analysis was done by mixed-effects analysis with Šidák post hoc tests for differences between groups compared to the 20 °C control group. *n* = 5 (10 °C), *n* = 4 (10 °C → 20 °C), *n* = 5 (20 °C), *n* = 4 up to day 14 and *n* = 3 from 30 dpi (25 °C) and *n* = 1 (30 °C). (**B**) Infarction fraction measured by semi-quantitative stereology on cryosections stained with anti-α-actinin (red), WGA (green) and Hoechst (blue). Histology could only be performed on animals that survived up til 60 dpi with *n* = 5 for 10 °C (all survived), *n* = 4 for 10 °C → 20 °C (all survived), *n* = 5 for 20 °C (all survived), *n* = 3 for 25 °C (3/4 survived) and *n* = 1 for 30 °C (1/6 survived). Statistical analysis performed by one-way ANOVA with Dunnett’s post hos tests for significant differences between the 20 °C control group and other groups. (**C**–**F**) Representative images of the immunofluorescence stain used for semi-quantitative stereology analysis presented in (**B**). Red areas are cardiomyocytes and green areas negative for α-actinin are remaining cryoinjury. Representative images from all included animals are shown in Supplementary Figure S2. * = *p* < 0.05, ** = *p* < 0.005 and *** = *p* < 0.0005. All error bars represent standard deviation.

With respect to the echocardiography data of the non-contracting fraction (two-way ANOVA with repeated measures and Dunnett’s post hoc tests), the 20 °C control group progressed as expected, having only a small remaining non-contracting fraction of 5.50 ± 1.83% at 30 dpi and 1.37 ± 0.37% by 60 dpi, which was significantly different from 4 dpi (12.79 ± 2.77%. *p* = 0.0122) (Figure 5A).

The 10 °C animals still had 15.19 ± 3.58% non-contracting fraction remaining at 60 dpi, which was not significantly different from 4 dpi (15.16 ± 3.53%) (*p* > 0.9999). The 10 → 20 °C group also showed no significant signs of regenerating by 60 dpi (10.75 ± 2.32%) when compared to 4 dpi (15.96 ± 2.56%) (*p* = 0.4573). However, once the animals were moved to 20 °C at 60 dpi, regeneration progressed and only 2.11 ± 0.38% non-contracting fraction remained by 60 + 14 dpi (*p* = 0.0263) and 0.33 ± 0.10% non-contracting fraction by 60 + 60 dpi (*p* = 0.0114) (Figure 5A). This demonstrated that the lack of regeneration at 10 °C was not due to an unfavorable and irreversible injury response.

When comparing the groups against the 20 °C control group (mixed-effects analysis with Šidák post hoc tests), both the 10 °C and 10 → 20 °C were also significantly different from the control at 30 dpi (*p* = 0.0098 and *p* = 0.0015 respectively) and 60 dpi (*p* = 0.0055 and *p* = 0.0216 respectively) (Figure 5A).

The 25 °C group had a non-contracting fraction of 9.99 ± 1.08% at 4 dpi. This was smaller than the 20 °C control group, but this was not significantly different at 4 dpi (*p* = 0.5313). When comparing to 4 dpi, the 25 °C group displayed significant regeneration by 60 dpi with a remaining non-contracting fraction of 3.18 ± 1.65% (*p* = 0.0233). At no point was the non-contracting fraction in the 25 °C group different from the 20 °C control (*p* = 0.9355 (14 dpi), *p* = 0.6134 (30 dpi) and *p* = 0.700 (60 dpi)) (Figure 5A). The one surviving cryoinjured animal at 30 °C had a non-contracting fraction of 0.76% at 60 dpi. Due to the high mortality in the 30 °C cryoinjured group, we cannot make any conclusions about cardiac regenerative rate at this temperature.

Assaying infarction fraction at 60 dpi (60 + 60 dpi for 10 → 20 °C group) by semi-quantitative stereology resulted in the same overall conclusion, with no statistically significant difference in infarction fraction comparing any of the groups to the 20 °C control, except for the 10 °C hearts, which still had a significantly higher remaining infarction fraction (one-way ANOVA with Dunnett’s post hoc tests against 20 °C control group, *p* < 0.0001 (10 °C), *p* = 0.2794 (10 → 20 °C) and *p* = 0.3258 (25 °C)) (Figure 5B–F and Supplementary Figure S2).

Overall, these results demonstrated that while heart regeneration was inhibited in a reversible manner at 10 °C, an equal or opposite effect was not seen at 25 °C, meaning an increase in temperature of 5 °C did not convey a regenerative disadvantage or benefit. At 30 °C, mortality meant that statistical analysis was not possible as only a single animal survived to 60 dpi, likely at least in part due to cardiac- and/or metabolic insufficiency after cryoinjury. Our findings on regeneration at different temperatures are strictly physiological, and future experiments are needed to uncover the specific mechanisms at play at the cellular and molecular level.

### 3.10. Animals Housed at 10 °C and 20 °C Gained Body Weight During the Regenerative Period, While Those Housed at Warmer Temperatures Failed to Do the Same

Although all animals followed a feeding regimen compensating for the expected changes in metabolic demand [28], only the 10 °C and 20 °C groups displayed weight gain throughout the regenerative period (two-way ANOVA with Dunnett’s post hoc tests) (Supplementary Figure S3). In the 10 °C group, there was a statistically significant weight gain from baseline at 14 dpi, 30 dpi and 60 dpi (*p* = 0.0363, *p* = 0.0028 and *p* = 0.022 respectively). In the 20 °C group, there was similarly a significant difference from baseline at all later time points (*p* = 0.0038 (4 dpi), *p* = 0.0028 (14 dpi), *p* = 0.0273 (30 dpi) and *p* = 0.0293 (60 dpi)). In the 25 °C group, there was no significant weight gain from baseline, and finally, in the 30 °C group, while no statistical test could be performed for only one animal, the single survivor lost weight after 14 dpi and onwards. It is possible that animals kept at higher temperatures underwent catabolism of proteins, fatty acids and glycogen stores in the tissues to sustain an increase in anaerobic metabolism, although we could not detect a significant difference in liver or fat body mass at the time of harvest (Supplementary Figure S4).

## 4. Discussion

The primary aim of this study was to determine how metabolism, cardiac function and cardiac regeneration were affected at different temperatures in the regenerative axolotl. As a general rule in ectothermic species, Q_10_ predicts that every 10 °C increase elevates metabolic demand by a factor of 2–2.5 [28].

### 4.1. Metabolic Effects in Uninjured Animals

Our measurements of oxygen consumption are measurements of aerobic metabolic rate, but do not account for anaerobic metabolism. In uninjured animals, O_2_ consumption rate scaled with temperature after acute exposure for 2 days (average Q_10_ of 2.76 across 10–30 °C) (Figure 4A), while after the 30-day acclimatization the relationship falls apart at the higher temperatures (Figure 3A). We cannot conclude whether this mismatch in the expected metabolic demand and O_2_ consumption after longer acclimatization is due to a major shift towards a greater degree of anaerobic metabolism (supported by an increase in plasma lactate (Figure 3C) and/or thermal acclimatization at the molecular level. Anaerobic metabolism in amphibians is highly temperature-dependent, but the capacity for anaerobic metabolism and lactate production in axolotls specifically is not known but may represent an important avenue of future study, with lactate found to have potential roles as a promoter of mammalian cardiomyocyte proliferation in vitro [37].

Importantly, anabolic processes to sustain growth (and thus regeneration) cannot be efficiently fueled by anaerobic processes and animals housed at 25 °C and 30 °C failed to maintain weight gain despite continuing to feed according to the expected Q_10_ adapted feeding regime (Supplementary Figure S3). Elevated plasma ketones may indicate an increase in fatty acid metabolism (Figure 4C); however, the mass of the liver and fat bodies after euthanasia as an indication of depletion of glycogen and fatty acid energy deposits, respectively, showed no significant changes compared to the 20 °C control in the 10 °C or 30 °C animals (two-way ANOVA with Dunnett’s post hoc tests, *p* ≥ 0.05) (Supplementary Figure S4).

Temperature deviations from the normal housing range could induce genuine mitochondrial dysfunction, for example, via thermal damage to respiratory chain complexes, altered membrane fluidity, and increased reactive oxygen species that impair oxidative phosphorylation. However, in ectotherms such as axolotls, downregulation of mitochondrial gene expression and oxygen consumption at low or high temperatures can also represent adaptive metabolic suppression to conserve energy in unfavorable conditions [38]. Distinguishing these mechanisms will require future experiments measuring both oxygen consumption and CO_2_, for which we did not have suitable equipment. In addition, direct measurements of mitochondrial capacity and impairment under different experimental conditions would be needed to fully elucidate these questions. Mitochondrial dysfunction and impairment of anaerobic fatty acid oxidation are also hallmarks of heart failure in humans [39].

### 4.2. Heart Function in Uninjured Animals

After the 30-day acclimatization, cardiac output in uninjured animals followed the expected Q_10_ relationship within 10–25 °C, while at 30 °C we did not see an equivalent increase, which could be indicative of cardiac insufficiency as the animals approached CT_lethal_. This likely also underpins the cause of high mortality, especially after injury, as cardiac reserve capacity becomes critical (Figure 3G–I).

Another interesting observation in relation to how the axolotl heart adapts to varying demands at different temperatures was that decreased cardiac output at 10 °C was predominantly achieved by decreased heart rate without changing contractility (VFAC). On the contrary, at 25 °C, the increased cardiac output was achieved by an increase in both heart rate and contractility (Figure 3G–I). Observations in other ectotherms suggest that increasing heart rate is more effective than increasing contractility for augmenting cardiac output at higher temperatures, with heart rate typically following a predictable positive correlation with temperature [40].

### 4.3. Pathological Signs at Higher Temperatures

Several indications point to pathological responses at 25 °C and 30 °C after the 30-day acclimatization in uninured animals. The breakdown of the expected Q_10_ relationship for both O_2_ consumption (Figure 3A) and cardiac function (Figure 3G–I), along with elevated LDH (sign of general tissue damage (Figure 3F)) and lactate (possible sign of elevated aerobic metabolism but also metabolic acidosis at higher levels [41] (Figure 3C)), suggests metabolic and cardiac dysfunction. This is particularly notable when comparing the effects of temperature changes after acute versus prolonged acclimatization, indicating that dysfunction emerged with prolonged thermal stress rather than from an inherent metabolic limitation. What specific tissues are contributing to the elevation of plasma LDH cannot be ascertained from our data, as this measurement is not specific to the heart, and inclusion of a cardiac-specific marker of tissue damage such as troponin would have been valuable in this regard. Elevated troponin would also strengthen the idea that the observed cardiac effects at elevated temperature represent some degree of cardiac insufficiency. Although we did not observe any behavioral changes that would be expected because of brain injury, it is also possible that high temperatures result in some level of neural toxicity, which could have an indirect effect on metabolism as well as cardiac function.

### 4.4. Regeneration at Different Temperatures

At 10 °C regeneration was effectively halted (Figure 5A,B), but this was not due to an alternative and ultimately irreversible response to injury. This is evident by the fact that animals moved from 10 °C to 20 °C quickly regulated their cardiac function to that of a normal 20 °C level and also went on to regenerate the injury over the next additional 60 days (Figure 5A,B). This shows that the T_opt_ for axolotl heart regeneration in particular is above 10 °C. Regenerative failure at 10 °C could, in large part, be due to a critical reduction in energy turnover and enzymatic activities at the subcellular level required for cell proliferation. Currently unknown mechanical cues that act to signal the presence of an injury and thus the need for a regenerative response could also be important. At 10 °C, heart rate was significantly reduced (Figure 3G) and mechanical forces like shear stress are likely reduced, although contractility was not affected, as evidenced by the non-significantly different VFAC compared to the control group (Figure 3H). In a porcine study, reducing heart rate had a positive effect on outcomes after myocardial infarction, largely via metabolic effects [42].

At 25 °C, regeneration progressed at a similar rate to that at 20 °C (Figure 5B,C). This could in part be a matter of cardiac reserve capacity and metabolic allocation of resources as the available oxygen and nutrients are directed towards maintaining the increased level of cardiac demand in an ectothermic species at higher temperature rather than supporting tissue synthesis, as such allocation can result in trade-offs with growth, reproduction, and energy storage that persist long after injury [43]. A positive effect on regenerative rate in salamanders with increased temperatures has been reported during limb regeneration in newts, though regenerating newts select intermediate temperatures (24–25 °C) rather than temperatures that would maximally accelerate regeneration, suggesting a balance between regenerative rate and metabolic costs [23]. Our observations of no regenerative benefit at 25 °C likely reflect a temperature relationship specific for cardiac regeneration.

The capacity for heart regeneration at 30 °C could ultimately not be determined due to the high mortality after cryoinjury (only one surviving animal out of six); however, an argument can be made that heart regeneration is not possible in vivo due to a systems-level failure making an injury incompatible with life because it requires a larger cardiac reserve capacity than what is available. This, however, does not exclude that the specific CT_max_ of cardiac regeneration may be higher in isolation, which could be explored in an in vitro setting where the metabolic demands of the organism are no longer a factor.

As predicted, the viable thermal range (CT_min_–CT_max_) narrowed after cryoinjury, evident by increased mortality at higher temperatures (Figure 2B). Without injury, axolotls tolerated 10–30 °C, but after cryoinjury CT_max_ decreased to 25–30 °C. with CT_lethal_ being around 30 °C. This narrowing suggests that cardiac reserve capacity, the heart’s ability to increase output beyond resting levels, becomes a limiting factor when thermal stress and injury are combined. While this study only provides indirect evidence that reserve capacity may limit heart regeneration, we propose it may in fact be an underappreciated determinant of regenerative ability. Cardiac reserve, which can vary 20-fold between highly impaired and fit states in humans [44], has not been examined across species in the context of cardiac regeneration. Such a multi-species study including cardiac stress tests and oxygen delivery capacity would be valuable in determining whether this represents a fundamental requirement for vertebrate heart regeneration. Further experiments are needed to understand the cellular and molecular mechanisms that regulate cardiac regenerative rate in response to temperature change. Temperature has organism-wide physiological effects on multiple systems, including but not limited to the endocrine system, such as stress responses, the immune system and enzymatic activity, all of which are likely to interplay and have some influence on regeneration.

### 4.5. Study Limitations and Further Effects of Temperature

It is a general limitation of our study that replicate numbers are low, meaning significant effects may not have been detected due to underpowered experiments. Furthermore, because the 30-day-acclimatized groups were fed one day prior to measurements, these metabolic parameters could have been affected by specific dynamic action, i.e., metabolic activity associated with digestion [29]. We tried to correct for this by normalizing the feeding schedule and using a longer fasting period in the 2-day acclimatization experiment, but did not do so for the 30-day acclimatization animals (although all measurements were taken in a postabsorptive state >24 h after last feeding). It is also possible that metabolic adaptation and pathological states could have affected energy turnover and/or nutrient absorption, which we have not accounted for.

While our cardiac function data indicate cardiac insufficiency at 30 °C, this would have been demonstrated more clearly if we had performed echocardiography throughout the acclimatization period and repeatedly at target temperature to evaluate the development of these variables in the same animals over time, especially in conjunction with cardiac injury markers and histology to evaluate tissue morphology and injury.

Our findings from PET imaging are also inconclusive. Although we have shown that glucose uptake was not the primary substrate involved in increased oxygen consumption in our experimental set-up, the most meaningful experimental conditions in terms of aeration and anesthesia are not clear. PET imaging of ectotherms is a relatively young field and it contains some challenges regarding, e.g., the selection of appropriate anesthesia and maintaining a stable oxygen supply and metabolic state [30]. Still, PET imaging could be valuable if a more detailed metabolic analysis were performed, assaying a broader range of metabolic substrates at different temperatures and oxygen levels.

It is also important to note that temperature has wide-reaching effects on animal physiology, all of which cannot be realistically evaluated in a single study. For instance, temperature has important implications for the immune system, which has been shown to be involved in the regenerative process [1]. However, we have previously demonstrated that axolotl heart regeneration is incredibly tolerant to both significant anti-inflammatory and pro-inflammatory stimuli [45].

Temperature also likely has widespread effects on molecular signaling elements that may be crucial in regulating the regenerative process. While heat shock proteins, stress hormones and hypoxia response elements like HIF1α are obvious candidates for such effects that warrant further studies, it is important to note that in an organism like the axolotl, every element of their biology has been evolutionarily shaped to function at their optimal living temperature (18–20 °C [33]). This was, for instance, exemplified by Villiard et al., showing that axolotl p53, which was essential for limb regeneration, became inactivated at higher temperatures [46]. However, our current work does not directly indicate that heart regeneration fails at higher temperatures, but rather that the animals cannot tolerate the injury as well due to limited cardiac reserve capacity.

In the case of intrinsic cardiac regeneration in the axolotl, we can conclude that this, on a physiological level, relies on sufficient cardiac reserve capacity that allows them to tolerate a substantial cardiac injury and yet deliver enough oxygen and nutrients via the systemic circulation. Yet decreasing their expected metabolic demand below levels at 20 °C did not convey any benefit, but rather inhibited regeneration, the exact mechanisms of which warrant further characterization. A likely explanation is simply that all metabolic, molecular and cellular processes are naturally slowed down at lower temperatures, for instance, as the activity of critical enzymes becomes so inhibited that cellular proliferation grinds to a halt. Furthermore, as increasing the temperature to 25 °C had no regenerative benefit, with some degree of mortality and ill effects, and at 30 °C, cryoinjury was not tolerated, it is apparent that higher metabolic demand and reduced cardiac reserve capacity limit the ability of axolotls to survive cardiac injury and thus undergo regeneration.

Importantly, successful cardiac regeneration depends on many elements as reviewed by others [47], such as a favorable immune response, the deposition of pro-regenerative extracellular matrix instead of fibrotic scar tissue at the injury site, angiogenesis and cardiomyocytes capable of re-entering the cell cycle. While some of these, especially the phenotype of cardiomyocytes, may be influenced by metabolic factors, some are likely also independent of metabolism.

## 5. Conclusions

We found that while healthy uninjured axolotls could be housed and survive at temperatures as low as 10 °C and as high as 30 °C, mortality after cryoinjury was nearly 100% at 30 °C and the animals underwent fundamental metabolic changes with signs of pathological tissue damage. Thus, while CT_max_ and T_lethal_ for axolotls were above 30 °C in an uninjured state, after cryoinjury, this fell to somewhere above 25 °C and below 30 °C. While mortality remained at 0% after injury at 10 °C, regeneration was highly reduced.

Overall, our findings supporting the hypothesis that cardiac regeneration can occur within a temperature range more narrow than CT_min_ to CT_max_, Importantly, while regenerative failure at colder temperatures likely result from slower metabolic turnover and enzymatic activity, the reason why regeneration is limited at higher temperatures, does not seem to be due to an inability for regeneration to occur per se, but rather the reduced cardiac reserve capacity and resulting mortality associated with cardiac injury.

## Data Availability

All raw data and analysis are available online.

## Supplementary Materials

The following supporting information can be downloaded at https://www.mdpi.com/article/10.3390/metabo16060414/s1: Figure S1: Mass-specific oxygen consumption rate of axolotls; Figure S2: WGA and α-actinin immunofluorescence and analysis; Figure S3: Body mass during the regenerative period; Figure S4: Liver and fat body mass.

## Abbreviations

The following abbreviations are used in this manuscript (in order of appearance):

CT_min_	Critical maximum temperature
CT_max_	Critical maximum temperature
T_opt_	Optimal temperature
T_lethal_	Lethal temperature
VFAC	Ventricular fractional area change
CO	Cardiac output
NCF	Non-contracting fraction
FDG	Fludeoxyglucose
PET	Positron emission tomography
MRI	Magnetic resonance imaging
LDH	Lactate dehydrogenase
WGA	Wheat germ agglutinin
CI	Cryoinjury
BPM	Beats per minute
dpi	Days post-injury
