# Supplementary material for: Temperature-Dependent Modulation of Cardiac Metabolism, Post-Injury Survival and Regenerative Rate in Axolotls
Source: Metabolites. 2026 Jun 13;16(6):414. doi: 10.3390/metabo16060414 (PMC13303739; doi:10.3390/metabo16060414)
Supplement: Supplementary file 1 [file metabolites-16-00414-s001.zip › metabolites-4276613-supplementary.pdf]

SUPPLEMENTARY MATERIAL FOR:

Temperature-Dependent Modulation of Cardiac Metabolism, Post-Injury Survival and Regenerative Rate in Axolotls

Anita Dittrich [1,*], Sofie Amalie Andersson [1], Aage Kristian Olsen Alstrup [2,3], Pernille Lajer Sørensen [1], Mette Irene Theilgaard Simonsen [2,3], Maibritt Hald Arildsen [1], Rasmus Roost Aabling [1] and Henrik Lauridsen [1,*]

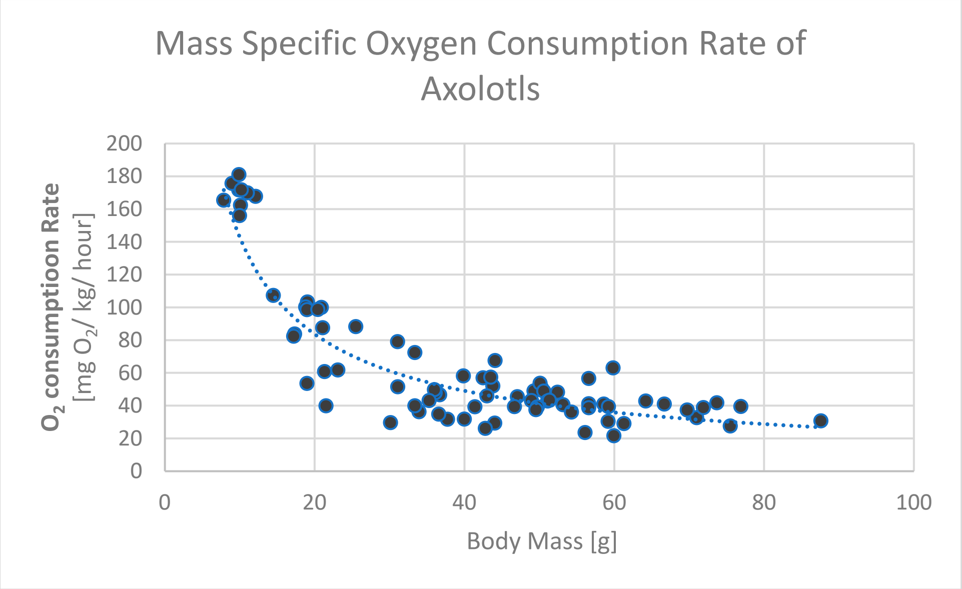

Figure S1. Mass specific oxygen consumption rate of axolotls. Oxygen consumption measured in axolotls of varying body sizes. Oxygen consumption was measured in a closed respirometry set-up as detailed in the methods section of the paper with the values plotted normalized to body mass and time spent in the respirometry chamber. The data presented here stem from the current study as well as additional published and unpublished work from our laboratory.

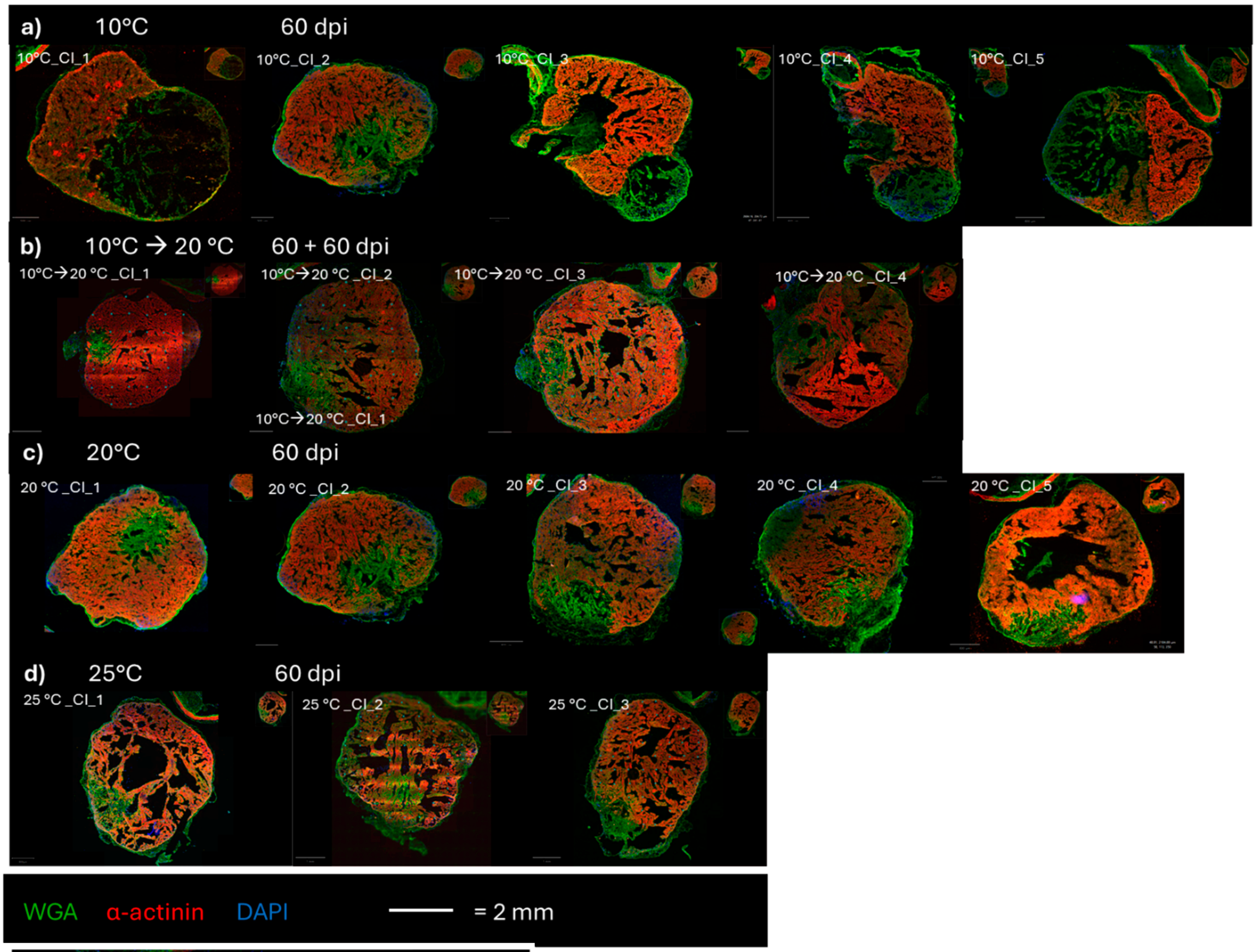

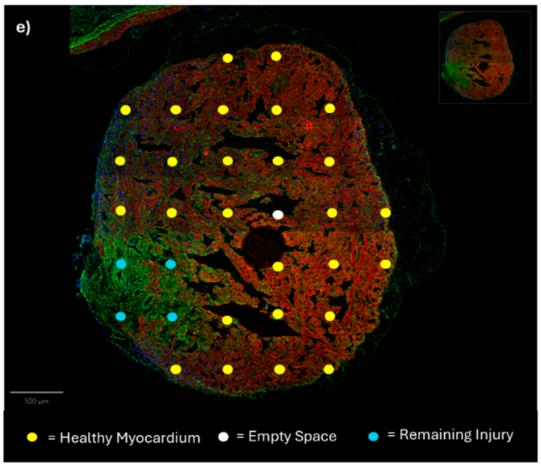

Figure S2. WGA and α-actinin Immunofluorescence and Analysis. a) Representative images from all 5 animals in the 10°C injury (CI) group. b) Representative images from all 4 animals in the 10°C →20°C injury (CI) group. c) Representative images from all 5 animals in the 20°C injury (CI) group. d) Representative images from all 3 surviving animals in the 25°C injury (CI) group. α-actinin is a marker for cardiomyocytes while WGA stains cell borders as well as collagen, this ventricular areas negative for α-actinin but positive for WGA is the remaining injury. e) example of a section analyzed in Qpath to calculate infarction fraction. Note that in 10C-CI-3 and -4 the opening to the atria with the associated valves are visible and α-actinin negative.

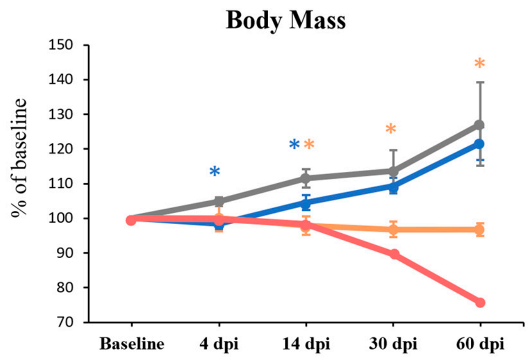

Figure S3. Body mass during the regenerative period. Includes all animals surviving until 60 dpi. Shown as relative to baseline starting weight, calculated individually for each animal before averaging. Error bars represent standard deviation. Color of asterisk indicates which group is statistically significantly different from the 20 °C control group. Blue = 10 °C (n = 5), grey = 20 °C (n = 5), orange = 25 °C (n = 3) and red = 30 °C (n = 1).

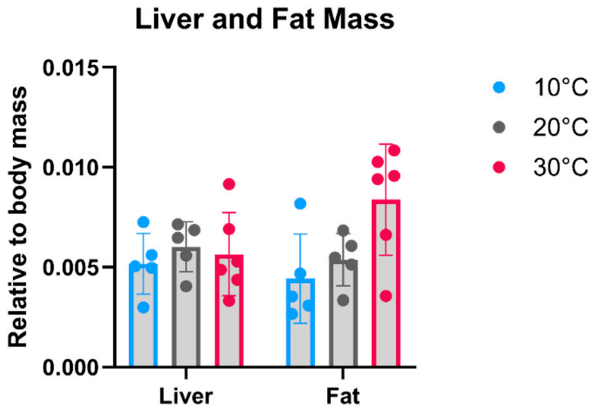

Figure S4. Liver and fat body mass. The mass of the liver and both fat bodies were measured after euthanasia in uninjured animals after 30 days of acclimatization and are shown here relative to the body mass of the animals. Neither the 10°C or 30°C animals had a liver- or fat-mass significantly differently of that of the 20°C control group (two-way ANOVA with Dunnett´s post hoc test, $p > 0.05$).
